# Gradient Boosting Machine Learning Model for Defective Endometrial Receptivity Prediction by Macrophage-Endometrium Interaction Modules

**DOI:** 10.3389/fimmu.2022.842607

**Published:** 2022-05-06

**Authors:** Bohan Li, Hua Duan, Sha Wang, Jiajing Wu, Yazhu Li

**Affiliations:** ^1^ Department of Minimally Invasive Gynecologic Center, Beijing Obstetrics and Gynecology Hospital, Capital Medical University, Beijing Maternal and Child Health Care Hospital, Beijing, China; ^2^ Department of Biochemistry and Molecular Biology, School of Basic Medical Sciences, Capital Medical University, Beijing, China

**Keywords:** immune infiltration, defective endometrial receptivity, infertility, Xgboost, machine learning

## Abstract

**Background:**

A receptive endometrium is a prerequisite for successful embryo implantation. Mounting evidence shows that nearly one-third of infertility and implantation failures are caused by defective endometrial receptivity. This study pooled 218 subjects from multiple datasets to investigate the association of the immune infiltration level with reproductive outcome. Additionally, macrophage-endometrium interaction modules were constructed to explore an accurate and cost-effective approach to endometrial receptivity assessment.

**Methods:**

Immune-infiltration levels in 4 GEO datasets (n=218) were analyzed and validated through meta-analysis. Macrophage-endometrium interaction modules were selected based on the weighted gene co-expression network in GSE58144 and differentially expressed genes dominated by GSE19834 dataset. Xgboost, random forests, and regression algorithms were applied to predictive models. Subsequently, the efficacy of the models was compared and validated in the GSE165004 dataset. Forty clinical samples (RT-PCR and western blot) were performed for expression and model validation, and the results were compared to those of endometrial thickness in clinical pregnancy assessment.

**Results:**

Altered levels of Mϕs infiltration were shown to critically influence embryo implantation. The three selected modules, manifested as macrophage-endometrium interactions, were enrichment in the immunoreactivity, decidualization, and signaling functions and pathways. Moreover, hub genes within the modules exerted significant reproductive prognostic effects. The xgboost algorithm showed the best performance among the machine learning models, with AUCs of 0.998 (95% CI 0.994-1) and 0.993 (95% CI 0.979-1) in GSE58144 and GSE165004 datasets, respectively. These results were significantly superior to those of the other two models (random forest and regression). Similarly, the model was significantly superior to ultrasonography (endometrial thickness) with a better cost-benefit ratio in the population.

**Conclusion:**

Successful embryo implantation is associated with infiltration levels of Mϕs, manifested in genetic modules involved in macrophage-endometrium interactions. Therefore, utilizing the hub genes in these modules can provide a platform for establishing excellent machine learning models to predict reproductive outcomes in patients with defective endometrial receptivity.

## Introduction

Defective endometrial receptivity (DER) is one of the prominent contributors to unexplained infertility (UI) and IVF failure. This disorder continues to frustrate and challenge the field of reproductive medicine due to several unknown etiology and few evidence-based diagnostic and treatment strategies. Emerging evidence indicates that the pregnancy success rate with assisted reproductive techniques is only 23%-45% for patients with unexplained infertility (1). In this view, defective endometrial receptivity is currently becoming a critical theory in the study of this disease. Estrogen and progesterone prepare the endometrium for pregnancy. Normal endometrial receptivity allows embryo attachment, implantation, invasion, and development of the placenta. However, defective endometrial receptivity may alter these processes. Research currently is underway to explore the causes of defective endometrial receptivity and biomarkers for the evaluation of endometrial receptivity (2). Some studies suggest an association of defective endometrial receptivity with uterine stem cell deficiency and enhanced cellular senescence. Such a correlation is thought to result in abnormal endometrial preparation for pregnancy, causing recurrent loss (3, 4). Another study on a gene microarray of endometrial receptivity defects found a close association between most of the biological alterations with immune processes (5).

Sex hormones regulate the interaction between immunocytes and the endometrium. Dramatic changes in the uterus during gestation involve immune acceptance of the fetus and placenta to ensure a successful pregnancy (6). A dramatic change to the relative proportions of leukocyte subpopulations *in utero* is the first histologically detectable maternal immunologic adaptation to the embryo (7). At the same time, the decidual macrophages manifest phenotypic plasticity to adapt to the local microenvironment. The decidual macrophages are characterized by an Mϕ1 (inflammatory) phenotype in the peri-implantation period (8). However, there exists a composite of Mϕ1 (inflammatory) and Mϕ2 (anti-inflammatory) decidual macrophages over the placental period, shifting to predominant Mϕ2 post-placentation, following potential stimulation by secretory factors (9). Also, subgroups beyond the scope of conventional phenotyping are present. While decidual macrophages may help avoid the occurrence of uterine infections in gestating women, compelling evidence suggests the increased relevance of endometrium-macrophage crosstalk in supporting normal placentation, given its contribution to implantation, placental development, immunoregulation, vascular remodeling, and tissue homeostasis (10). The matrix produces progesterone, prostaglandin E (PGE), and anti-inflammatory cytokines, including interleukin (IL) 10 and IL-4 (11). Progesterone potentially stimulates lymphocytes to produce anti-inflammatory cytokines and decidual dendritic cells (dDCs), which successively produce IL-10 and chemokine (C-C motif) ligand 17 (CCL17) (12). Immunoregulated dDCs induce the differentiation of T-helper type 2 cells (Th2) into decidual T-cells. Excepting altered immunocyte populations, studies have also revealed an association of dysfunctional immunoregulatory mechanisms with infertility and pregnancy loss. Immune dysregulation is considered among the most prevalent contributing factors for gestational interruptions in the peri-implantation period. Some of the vital contributors to pregnancy loss and infertility include decidual, placental, and fetal membrane infections (13). The endometrial infection can directly activate the decidual stroma to elicit either a proinflammatory or proapoptotic response. This subsequently alters the regional distribution, phenotype, and function of decidual immunocytes. Furthermore, the infection may impact pregnancy function and cell types of the tissues. As yet, an understanding of the pathways leading from infection to preterm labor is elusive despite mounting reports that innate immune receptors, including Toll-like receptors and Nod-like receptors may be essential factors (14).

Considerable evidence suggests a tight correlation of defective endometrial receptivity with the intrauterine immune microenvironment. However, the clinical value of such an association is largely underestimated. Presently, transvaginal ultrasound (TVUS) is the most common diagnostic test which measures the endometrium thickness. Despite the high sensitivity (99%) of TVUS, this method demonstrates an exceptional specificity, minimal at 3%. Like TVUS, imaging techniques, hysteroscopy, and endometrial biopsy have the disadvantage of insufficient accuracy (15). Emerging data shows that Endometrial Receptivity Array (ERA) guides clinical practice as it allows for the assessment of endometrial receptivity status through microarray analysis of 238 genes (16). However, it tends to necessitate higher lab levels for the detection and storage of specimens, limiting its popularity.

Herein, gene modules were constructed *via* weighted gene co-expression network analysis (WGCNA) to explore the application of the intrauterine immune microenvironment in clinical pregnancy prediction (17). The association of gene modules with integrated traits was evaluated. The predictors selected in this approach have representative biological structures and functions, allowing for successive selection of immune-endometrial interaction modules. Furthermore, this approach enables “dimensionality reduction” by selecting representative genes based on correlation coefficients. This reduces the number of indicators to be tested, thereby lowering the cost and difficulty in the establishment. Notably, the platform for assaying is a critical component that constrains the applicability of indicators. Each platform has a specific mechanism for data processing, with varying final performance. Machine learning models are optimal for handling such data. They integrate the test data and generate a comprehensive evaluation model while eliminating the instability caused by platform differences. Current evidence shows traditional regression, and random forest as the commonly applied machine learning models. The xgboost model, a kind of gradient boosting tree-structured model, has become a newcomer in machine learning due to its stability and flexibility. All of these models can integrate the data to emanate comprehensive evaluation indicators, which improve diagnostic effectiveness. However, significant differences in their data requirements and processing cannot be ignored. Random forest and xgboost models are more stable compared to traditional machine learning methods in diagnostic performance for different platforms. However, they pose more challenges in visualizing the impact of the data on the final diagnosis due to their “black box” calculations. The present study compares the pros and cons of the three calculation methods. Also, to evaluate the unique advantages of macrophage-endometrium interaction modules for endometrial assessment and their clinical utility, the calculation methods are compared to the prevailing ultrasonography using databases and clinical data, respectively.

## Methods

### Datasets and Patient Selection

All datasets were selected from chip microarrays. Four datasets (GSE58144, GSE71835, GSE92324, GSE165004) containing 107 DER patients and 110 controls were used to perform immune infiltration analysis, and GSE58144 for macrophage-endometrium interaction modules selection was applied for a machine learning predictive model. The GSE19834 dataset has a three replicate measure dataset telomerase-immortalized human endometrial stromal cell line (THESC) co-cultured with macrophages. The dataset is divided into 4 groups: vehicle-treated control, estradiol + progesterone, control + macrophage-conditioned medium, and estradiol + progesterone + macrophage-conditioned medium, including the microarrays of THESC and macrophages. The machine learning predictive model was validated using the GSE165004 dataset which includes 48 DER patients and 24 controls. Details of the microarrays are outlined in **Supplementary Table 1**.

**Figure 1:**
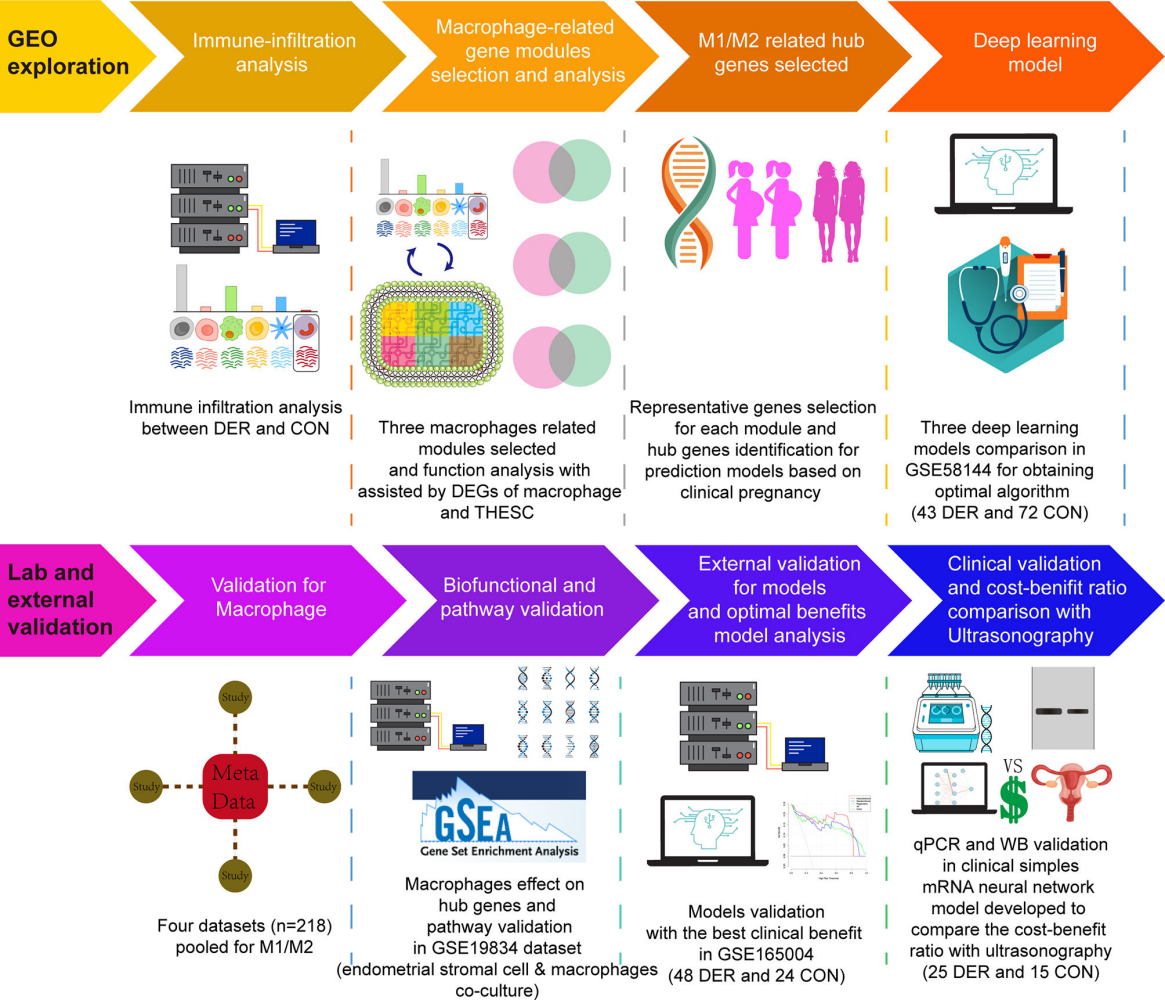
Schematic presentation of the study design. DER, defective endometrial receptivity.

Clinical samples were used to validate Mϕ1/Mϕ2, mRNA and protein levels. The Research Ethics Committee of the Beijing Obstetrics and Gynecology Hospital provided ethical approval for this study. Experiments were performed (under protocol number 2017-KY-082-02) following the Helsinki Declaration of 1975 (revised in 2013). Patients eligible for hysteroscopy were required to sign an informed consent before surgery and follow up for 1 year. Samples from endometrial biopsies were acquired at Beijing Obstetrics and Gynecology Hospital. The inclusion criteria: (i) Patients aged less than 45 years; (ii) Patients with sex hormone levels, including follicle-stimulating hormone, luteinizing hormone, testosterone, estradiol, and prolactin, within normal ranges; (iii). Patients without endometriosis, fibroids, active or a history of pelvic inflammatory disease, or other medical comorbidities (hyperprolactinemia, thyroid disease, etc.) after procedures. (iv). Patients are in or approaching the mid-secretory phase. Before starting the procedure, we first scraped a small amount of endometrium using a loop resectoscope without energizing to prevent cauterization of the specimen tissue and then powered up to perform the procedure after ensuring enough tissue be taken. Some patients with poor visual field exposure had their endometrium collected using electric suction aspiration prior to the procedure. Study participants in the control group (n=15) had successful clinical pregnancies, while those in the DER group (n=25) failed in pregnancy during the follow-up after procedures. Basic demographic characteristics for each group are presented in **Supplementary Table 2**. An overview of the study design is shown in **Figure 1**.

### Processing of Primary Datasets

The Biobase and limma packages (in R, version 3.6.2) were employed for pre-processing and normalization of microarray datasets based on raw data of the Agilent platform after the data had been converted to log (base 2). The RMA and normalizeBetweenArrays methods were applied for background correction and normalization, respectively. Annotation files for different microarray platforms were downloaded from the NCBI GEO database (18).

### Immune-Infiltration Analysis

Cell-type deconvolution was performed by CIBERSORTx (http://cibersortx.stanford.edu), an analytical tool developed by Newman et al. (19). This tool imputes gene expression profiles and estimates the abundances of immunocyte infiltration levels in mixed cell populations using gene expression data. The LM22 gene signature matrix for 22 immunocyte types was used. CIBERSORTx was run with batch correction and 100 permutations. Barplot and vioplot were constructed using the plot function (in R, version 3.6.2).

### Macrophage-Endometrium Interaction Modules Establishment

The network module was established *via* the WGCNA package (in the R environment, version 3.6.2) in the GSE58144 dataset (20). Data were filtered to select for modules associated with Mϕs alterations as described below. Through Pearson correlation analysis, all genes were ranked according to their association with Mϕ1/Mϕ2. Correlations between genes and Mϕ1/Mϕ2 were established at a cut-off of *p ≤* 0.05. By definition, module genes are highly connected (i.e., module genes tend to exhibit relatively high connectivity). A power of β=10 was selected according to scale-free topology criteria (R^2 =^ 0.85). In WGCNA, a soft threshold parameter, beta, of the power function was applied to ensure the best approximation of scale-free topology by the co-expression network (adjacency matrix). The weight threshold of the co-expression network was set to 0.03 to ensure that genes in the analyzed network are sufficiently correlated. The prcomp function (in R, version 3.6.2) was used for principle component analysis (PCA) and visualization of the modules.

### Enrichment Analysis of Functional Categories

The STRING v11.5 online tool (https://string-db.org/) was employed for functional enrichment analysis of the gene module that exhibited the highest association with endometriosis, identified post WGCNA analysis. For the enrichment analysis, Gene Ontology (GO) terms were used to evaluate functional categories and Kyoto Encyclopedia of Genes and Genomes (KEGG) pathways for genes associated with the module. Subsequently, Redundancy within lists of GO terms was reduced by the REVIGO algorithm, in which a simple clustering procedure with a concept similar to the hierarchical (agglomerative) clustering methods such as the neighbor-joining approach was performed (21). The GSE19834 dataset was used for differentially expressed genes (*P*<0.05) (DEGs) analysis and Gene-set enrichment analysis (GSEA) *via* “limma” and “ClusterProfiler” (22) packages in R. The Broad Molecular Signature Database (MSigDB v7.0) dataset in the KEGG (c2.cp.kegg.v7.0.symbols) were used as this database summarizes and presents specifically well-defined biological states and pathway processes. To estimate statistical significance, the GSEA program was run with 1,000 permutations. All genes were ranked based on the correlations between the selected genes and other genes.

### Quantitative Real-Time PCR Analysis (qRT-PCR)

Total RNA was extracted from each sample with RNAiso Plus kit (Takara Bio Inc., Shiga, Japan) and quantified using a NanoDrop™ One Spectrophotometer (Thermo Fisher Scientific Inc., Massachusetts, USA). cDNA was synthesized from 1 μg of total RNA per sample using the First-Strand cDNA Synthesis SuperMix Kit (AT301-3, EasyScript, China). The primers used in this study were designed by Sangon Biotech Co., Ltd. Shanghai, China. The primer sequences are shown in **Supplementary Table 3**. PCRs were performed in a LightCycler 480 PCR System (Roche, Germany) according to the recommendation of the manufacturer of SYBR Premix Ex TaqTM II (RR820A, Takara). The following PCR conditions were used: 95°C for 30 seconds for initial denaturation, followed by 35 cycles of 5 seconds at 95°C and 34 seconds at 60°C. The measurements were repeated three times, and the relative quantification was performed by the comparative CT (2^-ΔΔCT) method.

### Western Blot

Western blot analysis was performed as previously described. Equal amounts of a sample protein (50 μg) were electrophoresed onto an SDS-PAGE gel. After that, samples were transferred onto a nitrocellulose membrane. The membrane was blocked for 2 h at room temperature and incubated overnight at 4°C with the following primary antibodies: anti-MARF1 (1:500, proteintech, China), anti-dUTPase (DUT) (1:10,000, Abcam, USA), anti-RPS9 (1:500, proteintech, China), anti-β-Actin (1:10,000, ABclonal, China). Subsequently, appropriate secondary antibodies (1:5000, ABclonal, China) were incubated with the membrane for 1 h at room temperature. Blot bands were visualized with the ECL reagent (Western blotting Luminol Reagent, Santa Cruz Biotechnology, cat# sc-2048, USA). The measurements were repeated three times, and bands were selected to perform densitometry quantification using Image J software (Version 1.50b).

### Machine Learning Models Establishment and Statistics

Xgboost and Random Forest models were implemented *via* the Python package xgboost and Scikit-learn, respectively. The representative genes in each module were calculated with weights to verify their applicability in the machine learning model. The model are explained by Python package SHAP. The two models, including hub genes, were run in the GSE165004 dataset for verification. The splitting of the training and test sets was performed by the train_test_split package (in Python, version 3.8.8). Furthermore, the number of risk points corresponding to each weighted covariate used to construct the nomogram (23) in both GSE58144 and GSE165004 datasets were summed to calculate the diagnostic index. To test the efficacy of the predictive model, Z tests were used to determine the significance of the area under the receiver operating characteristic (ROC) curve (AUC) using the pROC package in R (version 3.6.2). The predictive values of hub genes and the machine learning model were evaluated based on sensitivity, specificity, Youden index (YI), positive predictive value (PPV), and negative predictive value (NPV).

A decision curve is valuable for the clinical model application evaluation by displaying the standardized net benefits estimates according to the probability threshold that classifies observations as “high risk”. The clinical impact curve is an alternative representation for the decision curve output. Herein, decision and clinical impact curves were generated using the DecisionCurve package in R, version 3.6.2 (24).

Cell proportions and single gene expression levels between the two groups were compared by the Wilcox test. Normally distributed continuous variables were analyzed using the Students t-test (in R, version 3.6.2). When the proportion of missing data is less than 10%, missing values were imputed using multiple imputation (mice package in R, version 3.6.2).

## Results

### Impact of Immune Infiltration Levels on Endometrial Receptivity

**Figure 2:**
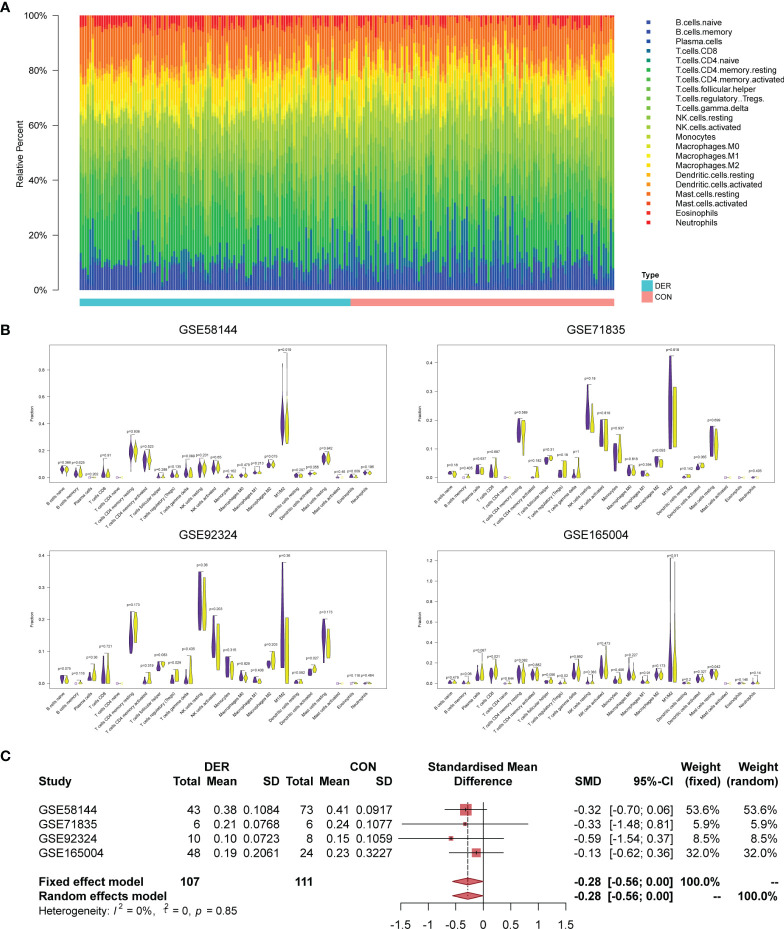
Immune infiltration analysis and Mϕ1/Mϕ2 balance. **(A)** Bar plots of 22 immunocytes in endometrial mixed tissue samples, including four datasets with 218 subjects. **(B)** Violin plots for immune cells in the DER and control groups. Purple color represents the control group while yellow represents the DER group. **(C)** Meta-analysis of four datasets for Mϕ1/Mϕ2. DER, defective endometrial receptivity; CON, control.

Immune infiltration levels of 22 immunocytes in endometrial mixed tissue samples from the four datasets were determined by the CIBERSORTx platform (**Figure 2A**). In the immune cell infiltration analysis of DER versus control for each dataset (**Figure 2B**), the Mϕ1/Mϕ2 maintained a consistent trend, showing a decrease in the ratio between Mϕ1 and Mϕ2 in the DER group. In addition, in the further meta-analysis, Mϕ1/Mϕ2 exhibited significant differences between the two groups in both the fixed and random effects models [-0.28 (95%CI -0.56-0)] (**Figure 2C**). Results demonstrated the infiltration variation of macrophages in different phenotypes is crucial for successful embryo implantation.

### Macrophage-Endometrium Interaction Modules Biofunction

**Figure 3:**
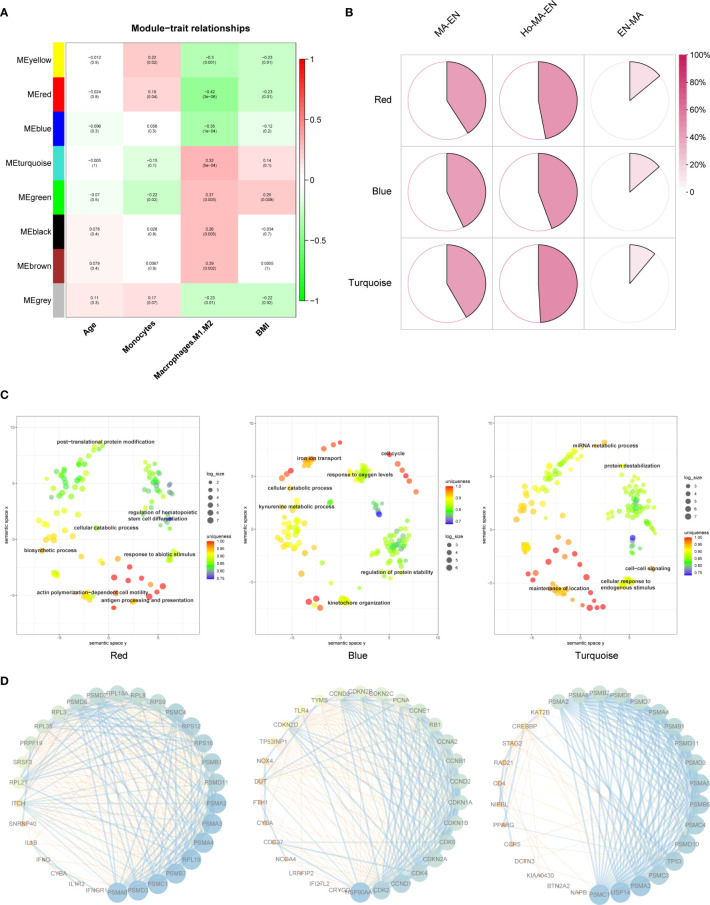
Mϕ1/Mϕ2-related WGCNA and module enrichment analyses. **(A)** Heatmap showing the average genetic significance of each particular module across clinical traits. **(B)** Percentage of GSE19834 DEGs within the modules. Percentages within modules are, from left to right, 40.9%, 47.0%, and 14.0% (red), 42.9%, 44.3%, and 13.7% (blue), 41.7%, 49.1%, 11.1% (turquoise), respectively. Ho-Ma-En=Endometrial cells in estradiol progesterone macrophage medium, Ma-En=Endometrial cells in macrophage conditioned medium, En-Ma=Macrophages in endometrial cell medium. **(C)** REVIGO plot of the red, blue, and turquoise modules (left to right). The scatterplot shows the cluster representatives in a two-dimensional space obtained by applying multidimensional scaling to the semantic similarities matrix of GO terms. **(D)** Protein-protein interaction (PPI) network of representative genes in each module. Each module has 30 representative genes selected according to their “degree”.

Mϕs related genes were clustered into different groups, referred to as modules. As described previous, the GSE58144 gene set comprised seven different gene modules with high topological overlap. The average genetic significance of a particular module was considered module significance (MS). Consistent with our previous study (22), three modules (red, blue, and turquoise) exhibited moderate correlations with Mϕ1/Mϕ2, that is, -0.42 (*p*=3×10^-6^), -0.35 (*p*=10^-4^), and 0.32 (*p*=5×10^-4^), respectively (**Figure 3A**). The integrated molecular profiles of the aforementioned modules were visualized using three-dimensional maps generated by the dimension reduction technique PCA. In **Supplementary Figure 1**, genes were grossly divided into symmetrical three subgroups by the modules depending on their relative up-regulation and down-regulation by references. This demonstrated that genes are characterized by unique profiles based on Mϕ1/Mϕ2 related modules.

The changes of endometrial stromal cells and macrophages after co-culture were evaluated in GSE19834 as a reference for modules biofunction. The interaction of endometrial stromal cells and macrophages can be reflected in the DEGs before and after the co-culture of the two. Afterward, the DEGs were intersected with genes within each of the modules. According to the proportion of the intersection genes within the module, we identified a high proportion of genes contributing to alterations in macrophages and endometrial cells after intercellular effects in all three modules, indicating that these are highly involved in constructing the macrophage-endometrium interaction microenvironment (**Figure 3B**). Subsequent enrichment analysis of genes within the module and the REVIGO visualization revealed that the biological functions of the red module were mainly enriched in processes of stress response and differentiation of immunocytes, including antigen processing and presentation, regulation of hematopoietic stem cell differentiation, response to abiotic stimulus, actin polymerization-dependent cell motility, among others. Regarding the blue module, genes were mainly enriched in cellular oxidative stress and cell cycle, such as iron ion transport, response to oxygen levels, cell cycle, were enriched. Regarding the turquoise module, genes were mainly enriched in macrophage-endometrium interactions, for example, cell-cell signaling, cellular response to endogenous stimulus, maintenance of location (**Figure 3C**). Additionally, the protein-protein interaction (PPI) network of representative genes contained within the three modules, based on their primary function, was shown in **Figure 3D**. Genes are ranked in order of their weighted node-degree in the combined network.

### Representative Genes and KEGG Pathways

**Figure 4:**
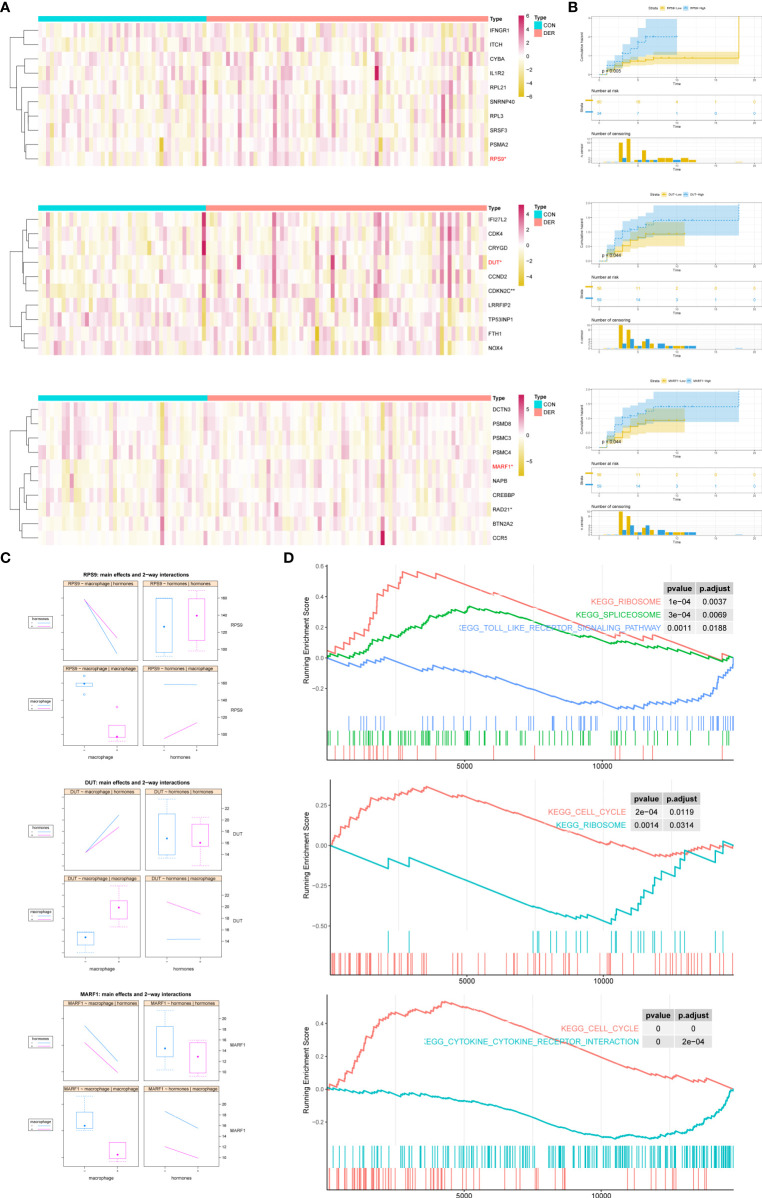
Representatives of the modules, and expression and functions of hub genes. **(A)** Heatmap of modules genes between DER vs. CON. In the classification column, red represents infertilities, whereas blue represents controls. **(B)** Cumulative risk curves of pregnant status analysis for hub genes. Log-rank *p* = 0.005 (RPS9), 0.044 (DUT), and 0.044 (MARF1) denotes the significance of hub genes. **(C)** Main effects and two-way interactions of hub genes. **(D)** GSEA plot of upregulated and downregulated KEGG pathways related to changes in hub gene expression levels. CON, control; DER, defective endometrial receptivity.

The “dimensionality reduction” was achieved by representative genes from three modules owing to the node-degree weight rank in the network and high consistency in expression among genes within the modules (*P*_membership in modules <_0.05). According to the enrichment biofunctions in string database, the representatives of the red module were macrophage-related genes (IL1R2, PSMA2, IFNGR1, ITCH, and CYBA), ribosome-related genes (RPS9, RPL3, and RPL21), and spliceosome-related genes (SNRNP40 and SRSF3). Representatives of the blue module were cell cycle-related genes (CDKN2C, CCND2, TP53INP1, and CDK4), oxidative stress-related genes (NOX4 and CRYGD), immune signaling-related genes (IFI27L2, LRRFIP2 and DUT), iron transport-related genes (FTH1). Representatives of the turquoise module were cell-cell signaling-related genes (CCR5, MARF1, PSMD8, and NAPB), cell cycle-related genes (CREBBP, PSMC4, PSMC3, DCTN3, BTN2A2, and RAD21). Furthermore, the Wilcox test revealed a significant association of RPS9, DUT, CDKN2C and MARF1 genes with infertility in the red, blue, and turquoise modules (**Figure 4A**). Furthermore, we explored the effect of hub genes on clinical pregnancy through Kaplan-Meier cumulative risk curves. Results demonstrated that RPS9, DUT, and MARF1 significantly improved the embryo implantation outcome for patients (**Figure 4B**). A two-way interactions analysis was performed to investigate further the effects of macrophages in combination with hormones on the three hub genes. Results suggested that macrophages significantly influenced the three hub genes (*P*_RPS9 _= 1.74×10^-5^, *P*_DUT_ = 0.0206, *P*_MARF1 _= 0.000187) without the significant interacting effects of hormones (*P*_RPS9 _= 0.15, *P*_DUT _= 0.41, *P*_MARF1 _= 0.593) (**Figure 4C**). Following single gene GSEA of the three hub genes in the GSE19834 dataset, along with the enrichment of genes within modules, results revealed the intersection of significant pathways as shown in **Figure 4D**: ribosome, splicesome, and toll-like receptor signaling (RPS9); cell cycle and ribosome (DUT); cell cycle and cytokine receptor interaction (MARF1).

### Construction and Validation of Machine Learning Models

**Figure 5:**
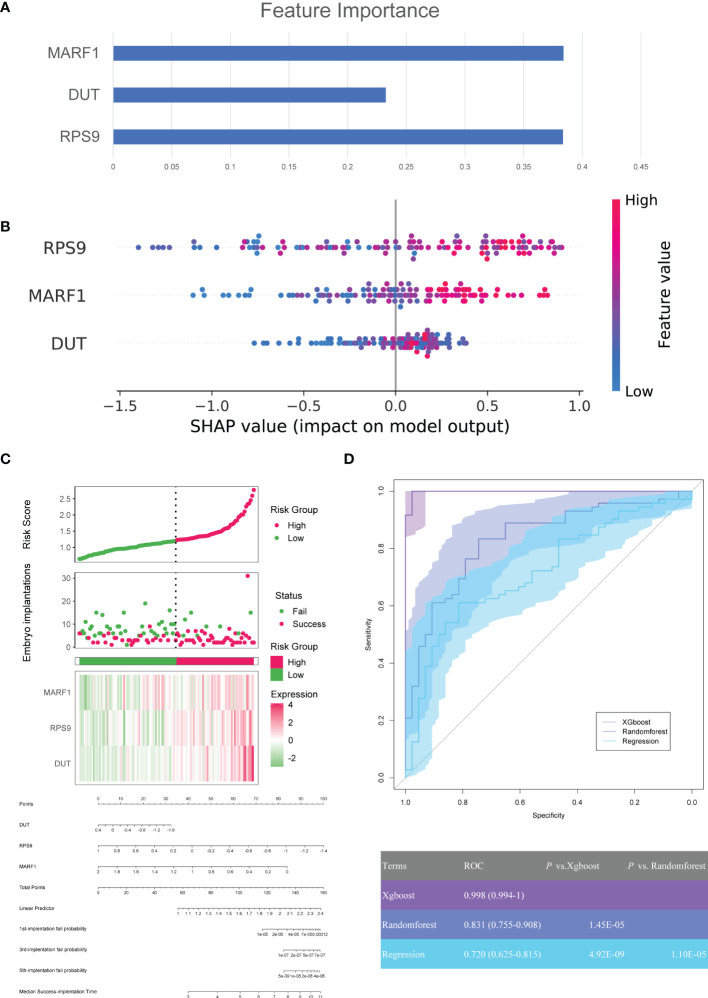
Construction of machine learning model for clinical pregnancy prediction. **(A)** SHAP values of each feature for random forests in modules. Each point represents a sample, and the figure shows the impact of features on the model’s outcome. **(B)** The weight matrix of hub genes in the xgboost model. Each column represents the weight of each feature to the model outcome in one permutation. **(C)** The predictive values of DUT, RPS9, MARF1, and the risk model were established based on logistic regression and visualized by a nomogram. **(D)** ROCs for machine learning models.

Machine learning algorithms (xgboost, random forest, and regression) were used to evaluate the predictive power of modules for defective endometrial receptivity. First, the weights of the genes within modules were evaluated separately for three machine learning methods. RPS9, DUT, and MARF1 showed high weights in three models. In the xgboost, random forest and the regression model, the three factors contributed similarly to the terminal outcome (**Figures 5A**–**C**). These findings demonstrate that the aforementioned genes hold great application prospects for model establishment.

In addition, three machine learning models, xgboost, random forest, and regression, were constructed for three genes in the GSE58144 dataset. After that, we plotted the diagnostic performance of the models in predicting embryo implantation outcomes with ROC (**Figure 5D**). The analysis demonstrated that AUCs of the three models were 0.998 (95% CI 0.994-1), 0.831 (95% CI 0.755-0.908) and 0.720 (95%CI 0.635-0.815), respectively. Meanwhile, the xgboost algorithm was significantly better compared to the other two in predicting embryo implantation outcomes (*P*=1.45×10^-5^ and 4.92×10^-9^).

**Figure 6:**
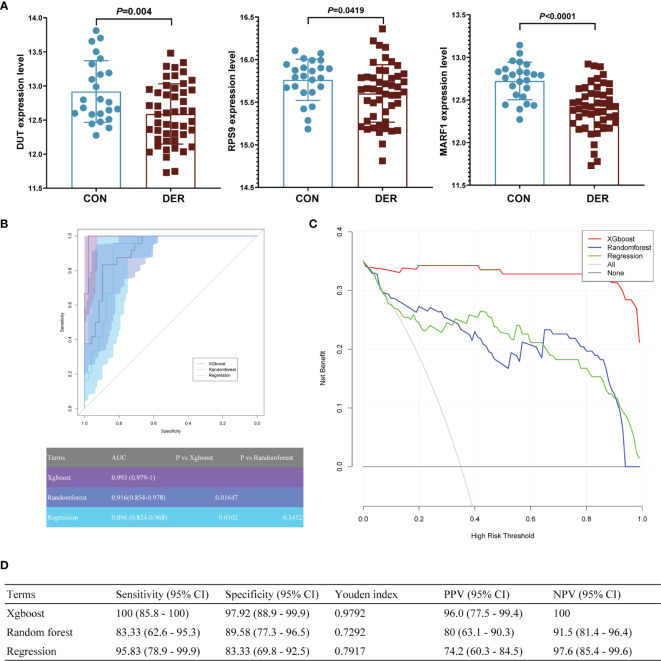
The clinical pregnancy prediction ability of the reference and machine learning models for in the external validation dataset. **(A)** mRNA expression of hub genes in GSE165004. **(B)** Receiver-operating-characteristics (ROC) curves. The corresponding value of the area under the receiver-operating-characteristics curve (AUC) for each model is shown in the table below. **(C)** Decision curve analysis. The X-axis represents the threshold probability for positive pressure ventilation outcome; Y-axis represents the net benefit. The net benefit of all machine learning models was larger over the range of clinical threshold compared to the reference model. **(D)** Sensitivity, specificity, Youden index, positive predictive value (PPV), and negative predictive value (NPV) of models.

Furthermore, the predictive model was validated in the GSE165004 dataset. The aforementioned hub genes were incorporated into machine learning models. Results showed that the expression levels of the three hub genes were significantly different between the two groups (**Figure 6A**), consistent with our previous research (22). According to ROCs, the xgboost model demonstrated superior diagnostic performance to the other two in the GSE165004 dataset. As such, the AUC of the xgboost model was 0.993 (95% CI 0.979-1), significantly better compared to that of the random forest and regression model (p=0.01647, and 0.0102) (**Figure 6B**).

According to the DCA of three prediction models, the net benefit for the xgboost model [0.343 (95%CI 0.321- 0.35)] was more prominent over the traditional regression model and random forest model [0.233 (95%CI 0.16-0.299) and 0.248 (95%CI 0.175-0.306), respectively] (**Figure 6C**). The findings demonstrate that the xgboost model is significant superior to the random forest model.

Results of sensitivity, specificity, YI, PPV, and NPV of the xgboost model were 100 (95% CI 85.8 - 100), 97.92 (95% CI 88.9 - 99.9), 0.9792, 96.0 (95% CI 77.5 - 99.4), and 100, respectively, superior to those of the other two models (**Figure 6D**).

### Clinical Benefits From Machine Learning Model

**Figure 7:**
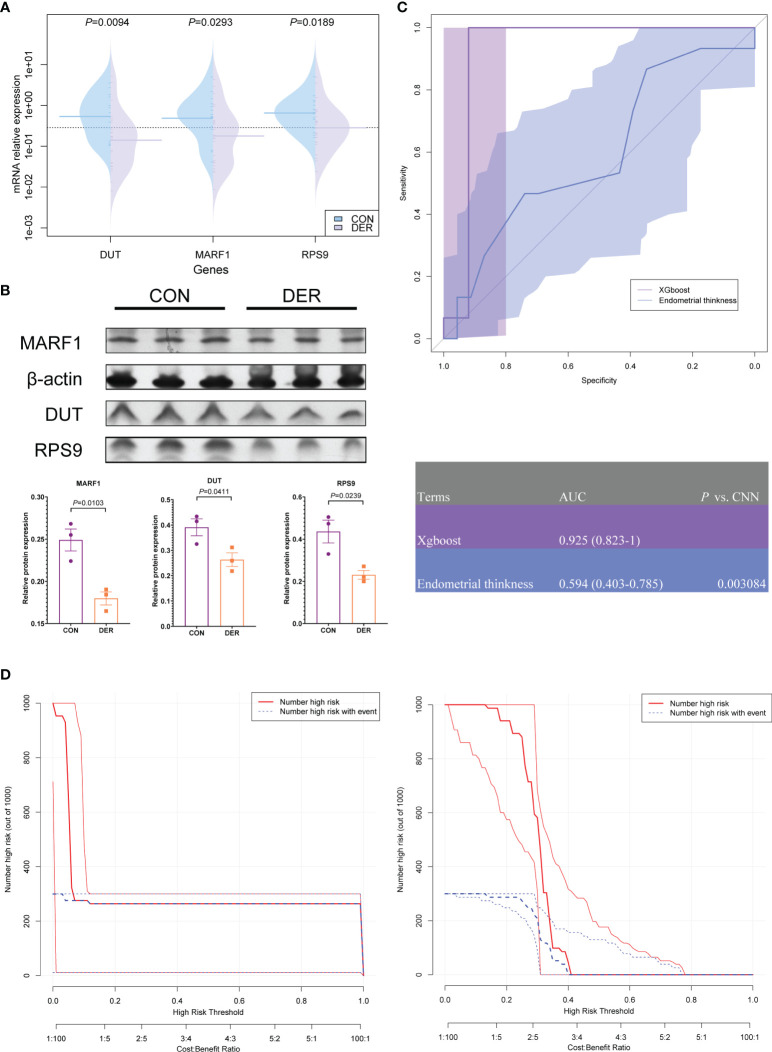
Hub genes and clinical validation and benefit analysis of the model. **(A)** Validation of mRNA expression of hub genes in DER (n=25) and control (n=15) groups. **(B)** Western blotting result of MARF1, DUT, and RPS9, normalized by β-actin (triplicates in each group). Bar graphs represent the ratio of densities of the respective protein bands and β-actin. Densitometric quantification graphs of blots are available in **Supplementary Figure 2**. **(C)** ROC of xgboost and ultrasound results of endometrial thickness. The corresponding value of the AUC for each method are presented in the table. **(D)** Clinical impact curve (CIC) of the xgboost model and endometrial thickness. The red curve (number of high-risk individuals) denotes the number of people classified as positive (high risk) by the model at each threshold probability; the blue curve (number of high-risk individuals with outcomes) denotes the number of true positives at each threshold probability.

The altered mRNA expression levels of RPS9, DUT, and MARF1 were verified in endometrial tissues. Results showed that the expression of all three genes (*P*=0.0094, 0.0293, and 0.0189) in DER patients was downregulated significantly compared to the controls (**Figure 7A**). In addition, the relative protein levels of the three were determined by Western blotting analysis (*P*=0.0103, 0.0411, and 0.0239) **Figure 7B**. We further analyzed MARF1 and found that the bands were mainly localized between 50-70 kDa (**Supplementary Figure 3**). Consistent with the previously reported LMKB, which is the human orthologue of MARF1 (25).

To verify the clinical efficacy of three hub genes for endometrial receptivity assessment, xgboost models were established based on the mRNA expression of 40 subjects. The models were compared to the commonly used ultrasonographic assessment for endometrial thickness. According to the ROC results, with the qPCR method, the model achieved a high detection level with an AUC of 0.925 (95% CI 0.823-1), significantly better than ultrasonography [AUC=0.594 (95% CI 0.403-0.785), *P*=0.003]. On the other hand, the sensitivity and specificity of ultrasonography were 86.67 (95% CI 59.5 - 98.3) and 34.78 (95% CI 16.4 - 57.3), respectively, when the cut-off value of >0.7 was applied; these results were inferior to the xgboost model [sensitivity 100.00 (95% CI 78.2 - 100.0); specificity 92.00 (95% CI 74.0 - 99.0)] (**Figure 7C**).

Clinical impact curve (CIC) analysis was performed to evaluate the clinical applicability of the xgboost model and ultrasonography (**Figure 7D**). Results of CIC analysis demonstrated that the xgboost model had a superior overall net benefit within the broad and practical ranges of threshold probabilities and impacted patient outcomes. The findings provide evidence that the xgboost model possesses significant predictive value and is superior to ultrasonography.

## Discussion

Altered immune microenvironment in the uterine cavity is not only an essential aspect of endometrial receptivity, but also crucial for successful implantation (26). However, application of immune-related factors is limited by the current research limitations and lack of effective clinical indicators. In the present study, we explored changes in levels of immune infiltration by pooling multiple datasets from 218 patients and detected macrophages, one of the predominantly altered immunocytes. Subsequently, we established macrophage-endometrium interaction modules and focused on their functions. Based on these, we establish corresponding machine learning algorithms models for clinically predicting pregnancy.

MФs play an essential role in pregnancy, where apart from participating in the decidua as a primary antigen-presenting cell, they also actively regulate early pregnancy trophoblast invasion, as well as tissue and vascular remodelling. Physiological events in the female reproductive system are inflammatory processes. MФs play a crucial role in initiation and progression inflammation, a phenomenon that occurs throughout all phases of the menstrual cycle and is scattered throughout the endometrium (27). Endometrial MФs increase during the early secretory phase, and continue to rise into the late secretory phase of the menstrual cycle with the distribution of sex hormones (estrogen and progesterone). Previous studies have also shown that estrogen and progesterone subtly regulate interactions between decidual immunocytes, endometrial cells, epithelial cells, and stromal cells (28). Physiological estrogen levels induce M-Ф proliferation and its positive and negative regulation of the production of CCL2, the M-Ф chemokine (29, 30). Elevated levels of pro-inflammatory cytokines, such as IL-6, leukaemia inhibitory factor (LIF), and tumor necrosis factor-alpha, in the endometrium, are features of early implantation. In addition, MФs and dendritic cells (DCs) have been shown to play an essential role in decidualization and implantation, where they regulate tissue remodelling and vascularization by producing a series of cytokines, chemokines, and enzymes. Furthermore, these molecules target the luminal epithelium and subsequently promote acquisition of endometrial receptivity. LIF, which is derived from MФs, regulates the structure of surface polysaccharides in epithelial cells during this process, thereby making the endometrium receptive (31). These processes were reflected in the three modules of the present study, which represented alterations in macrophages, endometrium, and cells cross-conductivity during mid-secretory endometrial receptivity.

Representative red module genes, as previously described, include genes related to macrophage, ribosome, and spliceosome. The module is mainly enriched in immunocytes’ stress response and differentiation progress, such as, antigen processing and presentation, regulation of hematopoietic stem cell differentiation, response to abiotic stimulus, and actin polymerization−dependent cell motility, among others. Changes in the intrauterine microenvironment can dramatically alter macrophage infiltration and polarization. Toll-like receptors (TLRs) are membrane-bound receptors that are expressed in innate immunocytes. TLR-mediated pathogen recognition stimulates rapid activation of innate immunity by eliciting production of pro-inflammatory cytokines and upregulation of costimulatory molecules. Furthermore, ribosomes are essential targets for epigenetic regulation in macrophages, and have been implicated in expression of long non-coding RNAs, regulatory mRNAs, and microRNAs. Notably, alterations in this pathway critical for macrophage infiltration and polarization alterations. Selective splicing (AS) of mRNA participates in regulation of immunocyte differentiation and activation, potentially regulating innate immune responses in macrophages. In fact, this process is inseparable from the spliceosome signaling pathway *via* regulating activation of the NF-κB signaling pathway (32). Overall, these evidences indicate that the red module is closely correlated with macrophage infiltration and polarization in the endometrium.

Blue representative genes included cell cycle, oxidative stress, and iron transport related genes. Generally, these genes play key roles in regulating biological processes, such as iron ion transport, response to oxygen levels, cell cycle focus on cellular oxidative stress and the cell cycle. On the other hand, the pathways regulating these functions include cell cycle, cytokine receptor interaction, and oxidative phosphorylation. Numerous studies have described the effect of iron ion metabolism and oxidative phosphorylation on endometrial cell biological functions. Notably, oxidative stress and iron ion overload commonly coordinate in the inflammatory response and metabolic abnormalities to affect the normal cycle and function of endometrial cells. Previous studies have shown that both ferrous and ferric ions can induce intracellular ROS formation *via* Fenton reactions, thereby causing formation of highly toxic hydroxyradicals, followed by accumulation of an oxidizing environment (33), which significantly affects regulation of the cell cycle. Previous studies have considered the hub gene, dUTpase, an essential enzyme that regulates nucleotide metabolism. Currently, several evidences have revealed its regulatory ability on immunocytes *via* the exosomal form. For example, Ariza et al. (34) demonstrated that dUTPase might mediate regulation of the cellular microenvironment *via* an intercellular signaling molecule that controls the innate immunity of human primary monocyte-derived macrophages through exosomal activation of Toll-like receptor (TLR)2, thereby leading to NF-kB activation and production of pro-inflammatory cytokines. Similarly, we also demonstrated the importance of ribosomes in endometrial receptivity within this module, which play key roles in macrophage and endometrial regulation. For example, Silvia Pérez-Debén et al. (35) evaluated endometrial receptivity-related pathways and found that the ribosomal pathway was the most relevant factor for endometrial fertility. The aforementioned mechanisms are all manifested by the interaction between macrophages and endometrium.

**Figure 8:**
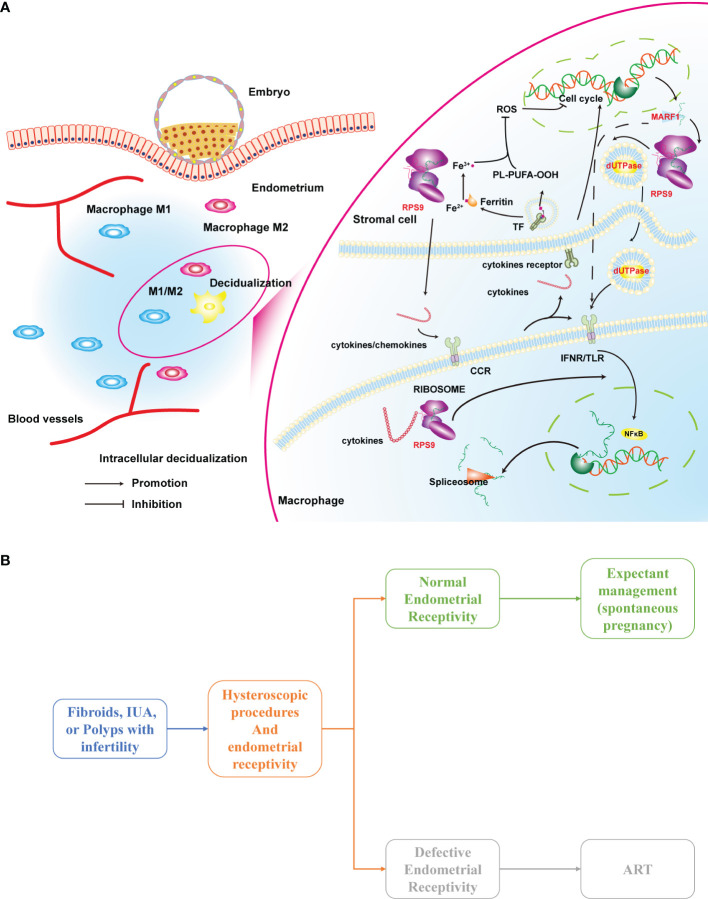
**(A)** A hypothesis on the mechanism of macrophage-endometrium interaction modules regulation on endometrial receptivity and **(B)** a scenario for clinical application of the defective endometrial receptivity prediction model. ART, Assisted reproductive technology.

Representatives in the turquoise module included cell-cell signaling, cell cycle related genes. These genes were functionally aligned with their gene modules and were highly enriched in biological processes that hinge on macrophage-endometrium interaction, such as cell−cell signaling, cellular response to endogenous stimulus, and maintenance of location, among others. In this module, the hub gene MARF1 is a P-body component thereof is a cellular structure that regulates cytoplasmic mRNA stability. In Bloch et al. (25), Limkain B (LMKB) is the human orthologue of MARF1, where the Ge-1-LMKB complex is located between 50 and 75 kDa. Interestingly, in this study, we found that the MARF1 bands showed high brightness shadows between 50-70 kDa (**Supplementary Figure 3**), similar to the mentioned study. This implicates that LMKB may be predominantly expressed in the human endometrium, which is an RNA-binding domain-containing protein that interacts with core-decapping proteins, suggesting that LMKB may also regulate mRNA stability (25). LMKB appears to have different functions in various cell types, that maintain the stability of the mRNA after transcription and may be associated with inflammatory response suppression. In addition, LMKB is expressed in human immunocytes where it plays a role in cellular response to type I interferon. Simultaneously, LMKB may be a relatively common target for cytoplasmic structures reacting with human autoantibodies, which has antigenic identity with MHC-I and is distributed in discrete cytoplasms, thus regulates activation of immunocyte-associated pathways (**Figure 8A**).

Next, we correlated DEGs under the interaction between endometrial stromal cells and macrophages in the GSE19834 with genes within the module with the aim of further validateing the enrichment functions of the three modules. When combined with results from enrichment analysis of genes and PCA within the modules, it was evident that the red, turquoise, and blue represents three independent aspects of macrophage-endometrial interaction function. In addition, interaction analysis of the hub genes demonstrated that the significant changes in the three genes due to macrophages did not interact with sex hormones, thus were suitable for incorporation into immune-based prediction models.

Although immunocyte infiltration and polarization play important roles in pregnancy, only a handful are available for reproductive prognosis. Notably, Diao et al. (26) developed an endometrial immunocyte-based score obtained encouraging results. Nevertheless, subjectivity of cell counts by immunohistochemistry and difficulty in standardization represent significant challenges for clinical application of the developed model. Moreover, immunocyte infiltration dynamics have been associated with some systematic errors. Therefore, in the present study, we anchored on immune-related factors as objective clinical predictors of pregnancy. Instead of the traditional dimension reduction, we adopted the WGCNA approach for selection of representative hub genes via, which has been shown to effectively reduce the number of indicators to be tested, making it accessible for clinical application. To overcome shortcomings associated with machine learning models without a clear interpretation of indicators, we first calculated the effect of three hub genes on reproductive prognosis under time accumulation by cumulative curves. We found that all three had a positive effect on amelioration of reproductive prognosis. In contrast, results from the xgboost used for subsequent model building based on both GSE58144 and GSE165004 datasets, revealed excellent diagnostic efficacy, significantly better than random forest and regression. We validated the model in clinical samples to affirm its clinical application. Previous studies have shown that ultrasonography, the most commonly used assessment method, guarantees a sensitivity of 99% and a specificity of 3% for endometrial thickness (>7 mm) (15). In comparison, our model had a sensitivity of 86.67 (95% CI 59.5 - 98.3) and a specificity of 34.78 (95% CI 16.4 - 57.3). Variation in the results can be explained by the relatively higher proportion of infertilities in our study than in previous. Compared to ultrasonography, xgboost models based on the macrophage-endometrial module improved the predictive efficacy significantly. Moreover, considering the clinical impact curve, it was evident that our xgboost model provides better clinical benefits than conventional ultrasonography. Furthermore, we used qRT-PCR to validate gene expression, and found that this cost-effective and universal technique can also befit clinical pregnancy prediction, owing to the possibility of real-time detection which enables timely investigation of clinical guidance of embryo implantation timing.

Despite the aforementioned advantages, this study also had limitations. Firstly, the 40 samples used for qRT-PCR validation to explore the clinical replicability of the model was small. In future, prospective studies using larger sample sizes are needed to validate our findings. Secondly, this was a one-center study. Therefore, further studies based on multiple centers are needed to affirm the predictive value and applicability of our model to other populations. Thirdly, although we cross-corroborated the related pathways of modules and hub genes by multiple datasets, we did not validate these findings using laboratory experiments. In short, based on our results, the consistency of validation, as analysed by different methods and datasets, was mutually confirmed.

Many infertility patients often suffer from fibroids, uterine adhesions, polyps, and endometriosis. These patients are recommended to try expectant management (spontaneous pregnancy) after the procedure without indications for assisted reproduction, which may lead many patients to miss the optimal window for conception by age or disease recurrence. Evaluation of endometrial receptivity intra- or post-operatively may be a preferable clinical indicator for these patients. As shown in **Figure 8B**, patients combined with defective endometrial receptivity can consider aggressive postoperative treatment such as assisted reproductive technology (ART). In contrast, for patients with normal endometrial receptivity, the attempt of spontaneous pregnancy is feasible.

In summary, exploring macrophage-endometrium interactions from a gene module perspective is a novel approach to investigate the mechanism underlying embryo implantation. Representative biological processes (immunocytes’ stress response and differentiation progress, cell−cell signaling, cellular oxidative stress and the cell cycle) and relevant pathways (ribosome, spliceosome, toll-like receptor signaling pathway, cell cycle, and cytokine receptor interaction) are essential components of endometrial receptivity. Furthermore, xgboost machine learning models could be an optimum approach for implementing macrophage-endometrium interaction module for clinical prediction of pregnancy-related complications, such as in patients with defective endometrial receptivity. Overall, this may contribute to a cost-effective and rapid ART timing assessment.

## Data Availability Statement

The original contributions presented in the study are included in the article/**Supplementary Material**. Further inquiries can be directed to the corresponding author.

## Ethics Statement

The studies involving human participants were reviewed and approved by the Research Ethics Committee of the Beijing Obstetrics and Gynecology Hospital (under protocol number 2017-KY-082-02). The patients/participants provided their written informed consent to participate in this study.

## Author Contributions

BL, Conception and design, Collection and assembly of data, Manuscript writing, Final approval of manuscript, Administrative support; HD, Conception and design, Manuscript writing, Administrative support, Final approval of manuscript; SW, Provision of study materials or patients, Manuscript writing, Final approval of manuscript; JW and YL Manuscript writing, Final approval of manuscript. All authors contributed to the article and approved the submitted version.

## Funding

This study was financially supported by the National Key Research and Development Program of China (2018YFC1004803) and Beijing Municipal Science & Technology Commission (No.Z211100002921015).

## Conflict of Interest

The authors declare that the research was conducted in the absence of any commercial or financial relationships that could be construed as a potential conflict of interest.

## Publisher’s Note

All claims expressed in this article are solely those of the authors and do not necessarily represent those of their affiliated organizations, or those of the publisher, the editors and the reviewers. Any product that may be evaluated in this article, or claim that may be made by its manufacturer, is not guaranteed or endorsed by the publisher.
