# Supplementary material for: Gradient Boosting Machine Learning Model for Defective Endometrial Receptivity Prediction by Macrophage-Endometrium Interaction Modules
Source: Front Immunol. 2022 May 6;13:842607. doi: 10.3389/fimmu.2022.842607 (PMC9120433; doi:10.3389/fimmu.2022.842607)
Supplement: Supplementary file 1 [file Table_1.docx]

**Supplementary Table 1. Overview of details for original studies**

| **GEO database** | **Region** | **Platform** | **Description** | **Sample size** |
| --- | --- | --- | --- | --- |
| GSE58144 | Endometrium | GPL15789 | Between 2006 and 2013, mid-luteal phase endometrial biopsies were taken in the natural cycle from two serial cohorts, each consisting of women with RIF, defined as ≥3 failed IVF/ICSI treatments or replacement ≥10 embryos without the occurrence of pregnancy and controls. All subjects had undergone IVF/ICSI treatment in two tertiary hospitals. The control group had all conceived within the first two cycles of ICSI or first three cycles of IVF treatment, and were therefore considered unlikely to have an endometrial factor. Microarray profiling and subsequent analysis sought to identify a gene expression signature predictive of RIF. | 43(DER) vs. 72(CON) |
| GSE165004 | Endometrium | GPL16699 | All patients involved in this prospective cohort study were recruited from Istanbul University School of Medicine between August 2014 and August 2015. Three cohorts (fertile controls, patients with RPL and UI) were studied. None of the patients has received a prior infertility treatment and were not under a current treatment. The first cohort comprised fertile control patients who presented to our gynecology department for well woman examinations. The inclusion criteria were regularly cycling women aged under 35 years with at least one live birth, no history of infertility/treatment, no previous miscarriages and no associated gynecologic (endometriosis, fibroids, active or history of pelvic inflammatory disease) or other medical comorbidities (hyperprolactinemia, thyroid disease etc). The remaining cohorts constituted patients who presented to our in vitro fertilization (IVF) unit. The second cohort included patients with RPL with no history of successful pregnancies. The inclusion criteria for this group were regularly cycling women aged under 35 years with at least two consecutive pregnancy losses of 20 weeks or less, normal follicle-stimulating hormone (FSH), luteinizing hormone (LH), estradiol (E2), prolactin (PRL), and thyroid-stimulating hormone (TSH) levels at day 2-3, normal uterine cavity shape and size, and bilateral tubal patency observed on hysterosalpingogram, no mutations detected in Factor V (Leiden) and prothrombin gene analysis, normal antithrombin III, protein C and S activity, negative results for lupus anticoagulant evaluation, cardiolipin antibody (IgM and IgG), and beta2-glycoprotein antibody (IgM and IgG) and normal karyotype. Their partners have normal spermiogram results and normal karyotype. | 48(DER) vs. 24(CON) |
| GSE71835 | Endometrium | GPL10558 | Implantation failure cases with unexplained infertility (having normal hormones and endometrium with 8 mm and above, triple layered) were compared with oocyte donors as healthy proven fertile controls under the influence of long protocol of ovarian stimulation during IVF. | 6 (DER) vs. 6 (CON) |
| GSE92324 | Endometrium | GPL10558 | Implantation failure cases with unexplained infertility (having normal hormones and endometrium with 8mm and above, triple layered) were compared with oocyte donors as healthy proven fertile controls under the influence of long protocol of ovarian stimulation during IVF. | 8 (CON) vs. 10 (DER) |
| GSE19834 | Endometrial stromal cells and macrophages | GPL2895 | Manipulations of biological samples and protocols used: THESC cells were grown in culture in phenol red-free DMEM/F12 medium supplemented with 1% ITS+, 2% charcoal/dextran treated fetal bovine serum, 1% penicillin/streptomycin, and 500 ng/ml puromycin. The cells were treated for 8 days with vehicle (0.1% ethanol), estradiol (10-8 M) plus progesterone (10-7 M medroxyprogesterone acetate), vehicle + macrophage-conditioned medium, or estradiol (10-8 M) + progesterone (medroxyprogesterone acetate 10-7 M) + macrophage-conditioned medium. On day 6, serum was removed from the culture medium of all cells. On day 7, half of the cells were treated with macrophage-conditioned medium for 24 hours. The cells were harvested and RNA extracted on day 8.  The experiment was repeated with three different passages of cells (passages 9, 11, and 13) to yield an experimental n of 3. The four groups of cells were vehicle-treated control, estradiol + progesterone, control + macrophage-conditioned medium, and estradiol + progesterone + macrophage-conditioned medium. | THESC ENDOMETRIAL CELLS CONTROL×3  THESC ENDOMETRIAL CELLS ESTRADIOL+PROGESTERONE×3  THESC ENDOMETRIAL CELLS VEHICLE+MACROPHAGE-CONDITIONED MEDIUM×3  THESC ENDOMETRIAL CELLS ESTRADIOL+PROGESTERONE+MACROPHAGE-CONDITIONED MEDIUM×3  MACROPHAGES CONTROL×3  MACROPHAGES+THESC-CONDITIONED MEDIUM×3 |

**Supplementary Table 2. Overview of the demographics and other characteristics of the recruited patients**

| Parameter | CON  N=15 | DER  N=25 | *P* |
| --- | --- | --- | --- |
| Age, mean (SD) | 34.07 (3.19) | 35.67 (5.22) | *P*= 0.43 |
| Irregular menstruation, n (%) | 1 (6.7) | 1 (4) | *P*=1 |
| BMI, mean (SD) | 23.48 (3.75) | 21.44 (5.86) | *P*=0.37 |
| Gravidity, median (range) | 1 (0, 3) | 1 (0, 7) | *P*= 0.68 |
| Parity, median (range) | 1 (0, 2) | 0 (0, 2) | *P*=0.72 |
| Hemoglobin, mean (SD) | 125.53 (8.63) | 125.08 (14.94) | *P*=0.91 |
| Uterine volume, mean (SD) | 63.49 (18.28) | 64.47 (33.83) | *P* =0.57 |
| Endometrial thickness, mean (SD) | 11.2 (4.50) | 9.83 (4.76) | *P*=0.34 |

**Supplementary Table 3. Specific primers used in qRT-PCR analysis.**

| **Gene** | **Sequence (5′-3′)** | |
| --- | --- | --- |
| **DUT** | Forward | 5′-GGTGATCGAATTGCACAGCTC-3′ |
|  | Reverse | 5′-TGAACCCCTTTCGGTGTCATC-3′ |
| **RPS9** | Forward | 5′-GAAATCTCGTCTCGACCAAGAG-3′ |
|  | Reverse | 5′-GGTCCTTCTCATCAAGCGTCA-3′ |
| **MARF1**  **(LMKB)** | Forward | 5′-ACCCTCCACTTCGCCAATG-3′ |
|  | Reverse | 5'-CTTTGCGAGTCTAACAGTGCG-3′ |
| **ACTB** | Forward | 5′-CATGTACGTTGCTATCCAGGC-3′ |
|  | Reverse | 5′-CTCCTTAATGTCACGCACGAT-3′ |
