# Supplementary figures and images for: Gradient Boosting Machine Learning Model for Defective Endometrial Receptivity Prediction by Macrophage-Endometrium Interaction Modules
Source: Front Immunol. 2022 May 6;13:842607. doi: 10.3389/fimmu.2022.842607 (PMC9120433; doi:10.3389/fimmu.2022.842607)

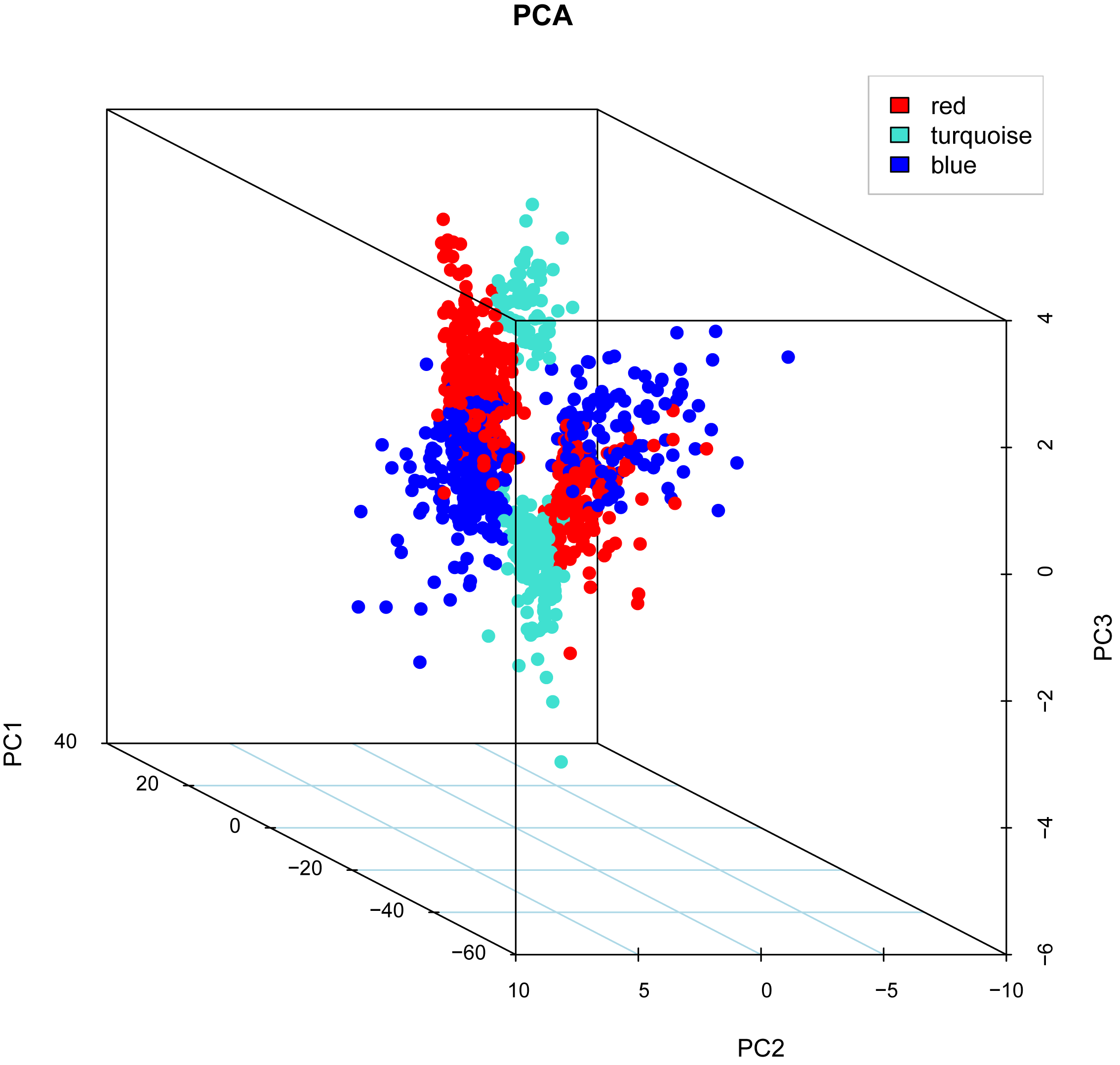

Supplement: Supplementary file 2 [file Image_1.tif]

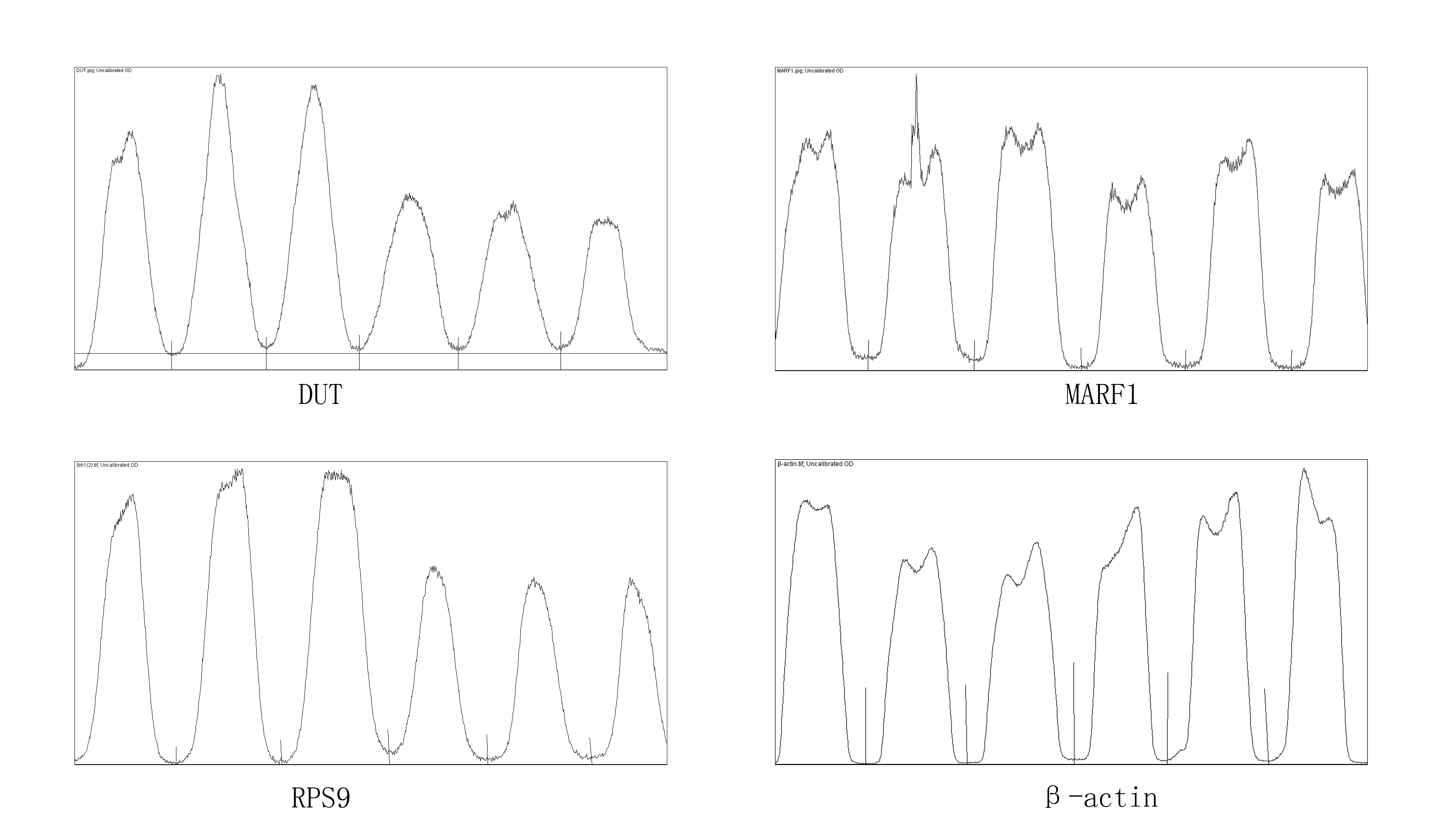

Supplement: Supplementary file 3 [file Image_2.tif]

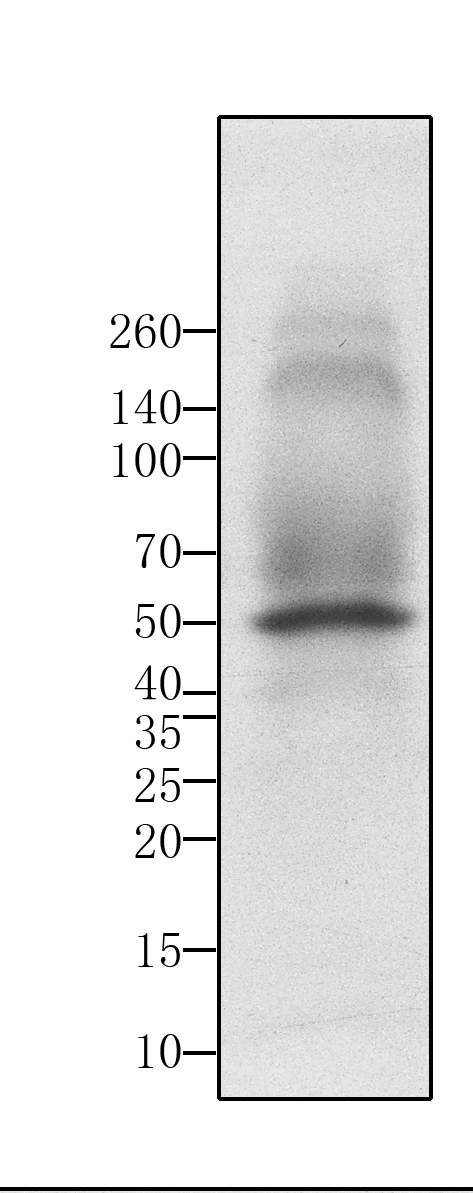

Supplement: Supplementary file 4 [file Image_3.tif]
